# A Mass Spectrometry Approach Reveals Fatty Acid Isomerism in Tomato Cold Tolerance

**DOI:** 10.1002/advs.202500175

**Published:** 2025-08-04

**Authors:** Leelyn Chong, Hengxue Shi, Qirui Yu, Xiaoning Shi, Zhaoxing Jia, Ziyun Dong, Yu Xia, Yingfang Zhu

**Affiliations:** ^1^ State Key Laboratory of Crop Stress Adaptation and Improvement School of Life Sciences Henan University Kaifeng 475001 China; ^2^ MOE Key Laboratory of Bioorganic Phosphorus Chemistry & Chemical Biology Department of Chemistry Tsinghua University Beijing 100084 China; ^3^ Sanya Institute of Henan University Sanya Hainan 572025 China; ^4^ Key Laboratory of Quality and Safety Control for Subtropical Fruit and Vegetable Ministry of Agriculture and Rural Affairs College of Horticulture Science Zhejiang A&F University Hangzhou Zhejiang 311300 China

**Keywords:** cold stress, FAD, fatty acids, HY5, isomers, Paternò‐Büchi (PB) reaction

## Abstract

Plants can adapt to environmental fluctuations through modulating their fatty acids (FAs) dynamically. In this study, an enhanced mass spectrometry approach is utilized to uncover an unexplored landscape of FAs and FA isomers that are critical for cold tolerance in tomato. This technology integrates N‐(4‐aminomethylphenyl) pyridium derivatization of FAs, charge‐tagging Paternò‐Büchi (PB) photochemical reaction to identify carbon–carbon double bond (C═C) positions and reversed‐phase liquid chromatography coupled with tandem mass spectrometry to achieve efficient detection of FAs and their C═C location isomers. Several saturated FAs, unsaturated FAs and their C═C location isomers are revealed to contribute to the cold tolerance of *elongated hypocotyl 5* (*slhy5*) and *fatty acid desaturase* (*slfad*) mutant plants. RNA‐sequencing analysis and dual‐luciferase reporter assays further demonstrate that *Sl*HY5 can modulate the expression of *Sl*FAD2 genes under cold stress, regulating FA desaturation. The application of FA isomers to the leaves of *slfad* mutants partially rescues their cold sensitivity, presenting the practical implications of the study. The study thereby highlights the importance of considering isomeric variations in FAs when investigating plant physiology and stress responses. Furthermore, this methodology sets a valuable precedent for future investigations aimed at unraveling the intricate metabolic networks that govern plant stress adaptation.

## Graphical abstract

An enhanced mass spectrometry approach reveals fatty acids (FAs) and FA isomers that are critical for tomato cold tolerance. Applying specific FA isomers can partially rescue cold sensitivity in tomato mutant plants, highlighting the importance of FA isomers in plant cold stress adaptation.

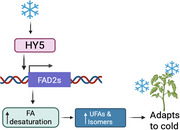

## 1 Introduction

Cold stress in plants is a significant environmental hazard that can detrimentally impact plant growth, development, and overall productivity.^[^ ^1^, ^2^, ^3^, ^4^ ^]^ With climate change leading to increased frequency and intensity of cold stress in many agricultural regions, it becomes critical to identify factors that can enhance crop's resilience to cold. Some plant species are reported to exhibit strong adaptability to cold stress due to specific genetic traits while others may not respond favorably even when exposed to similar conditions.^[^ ^5^, ^6^ ^]^ The interactions between genetic, environmental, and physiological factors in the cold stress response of plants are complex and not fully comprehended.^[^ ^7^, ^8^ ^]^ Therefore, it is important to untangle these intricate relationships so that a more comprehensive framework of cold‐resilient crops can be achieved to engineer crops that can survive and thrive in suboptimal temperatures. Technologies such as CRISPR gene editing combined with genomic and transcriptomic analyses represent a transformative approach to identify key genes responsible for cold tolerance in plants. ^[^ ^6^, ^9^, ^10^, ^11^, ^12^, ^13^ ^]^ While these innovations provide valuable insights, it is important to integrate them with knowledge and methodologies from other scientific disciplines to achieve more robust outcomes and build a holistic understanding of cold stress response in plants.

Fatty acids (FAs) are the building blocks of lipids and they play crucial roles in plant stress response, serving as versatile signaling molecules that help plants adapt to various environmental challenges.^[^ ^14^ ^]^ When plants encounter stressful conditions, such as extreme temperatures, drought, or pathogen attacks, their cells rapidly initiate a cascade of biochemical reactions that involve the mobilization and modification of FAs. These FAs act as dynamic messengers, triggering a series of defense mechanisms that allow the plant to mount an effective response.^[^ ^15^, ^16^, ^17^ ^]^ For instance, certain FAs can activate the production of protective enzymes, stimulate the synthesis of stress‐related hormones, or even directly inhibit the growth of invading pathogens.^[^ ^18^, ^19^ ^]^ Moreover, the composition and distribution of FAs within the plant cell membranes can undergo rapid changes, altering the membrane fluidity and permeability to better withstand stressful conditions. This flexibility in FA‐mediated signaling enables plants to rapidly perceive, interpret, and respond to a wide range of environmental stressors, enhancing their overall resilience and survival.^[^ ^20^ ^]^ It is evident that the interplay between FAs and plant stress response is intricate, requiring a multidisciplinary approach to fully unravel.

Studying the lipid composition of plants can be an incredibly complex and challenging endeavor due to the sheer diversity and variability of the FAs that make up these organic compounds. At the core, plant lipids are primarily composed of long‐chain unsaturated FAs (UFAs), which contain one or more carbon‐carbon double bonds (C═C) in their hydrocarbon structure. This unsaturated nature not only gives these FAs a more fluid, flexible structure compared to the rigid, saturated FAs (SFAs) found in many animal fats^[^ ^21^ ^]^ but also can exist in multiple structural forms known as isomers. Fatty acid isomers, including location isomers with varying C═C bond positions, geometric isomers with double bond configurations (*cis* or *trans*), and chain‐modified FAs with branched or hydroxyl groups, exhibit diverse chemical properties and biological activities. These isomers are critical for understanding lipid diversity and their impact on plant and human health, as they influence membrane fluidity, nutrient absorption, and cellular signaling pathways.^[^ ^22^, ^23^ ^]^ Analyzing and differentiating between these lipid isomers found in plant tissues is a challenging task, requiring advanced analytical techniques to accurately identify and quantify complex lipid profiles. Further complicating matters, the relative abundance of FA isomers can vary widely depending on the specific plant species, the growing conditions, and the plant tissues being examined. All of these variables must be well‐controlled to gain a comprehensive understanding of the nuanced lipid chemistry occurring within the plant kingdom.^[^ ^24^, ^25^ ^]^ Nevertheless, the inherent structural diversity and isomeric complexity of plant lipids present both an exciting challenge and an opportunity for us to study these important biomolecules.

In our current work, we employed an analytical method that has not been applied in the field of plant lipidomics to examine the dynamics of FAs, including SFAs, UFAs, and their isomers in the cold stress response of tomato (*Solanum lycopersicum*) plants. Our approach applied N‐(4‐aminomethylphenyl)pyridium (AMPP) derivatization and reversed‐phase liquid chromatography‐tandem mass spectrometry (RPLC‐MS/MS) to profile FAs at the chain composition level. In concert with this methodology, we incorporated a charge‐tagging Paternò‐Büchi (PB) reaction, a photochemical derivatization process involving 2‐acetyl pyridine (2‐acpy) to target the C═C bonds within unsaturated lipids. The logistics behind the PB reaction center around a concerted [2+2] cycloaddition mechanism, in which the carbonyl group of 2‐acpy reacts with the double or triple bond of a lipid to form a cyclopropane ring. This reaction requires the presence of UV light or other activation methods to initiate the [2+2] cycloaddition process and create a four‐membered oxetane ring. When the oxetane ring is further processed in MS using ion activation methods such as collision‐induced dissociation (CID), a unique fragmentation pathway emerges. The preferential breakdown of the oxetane ring produces diagnostic ions that are unique to the initial C═C bonds of reactants. This unique coupling with RPLC‐MS/MS enabled us to identify and quantify FA C═C location isomers.^[^ ^26^, ^27^, ^28^ ^]^ Using this methodology, we gained insights into the existence and distribution of various FAs and FA C═C location isomers. By combining this advanced analytical framework with other analyses, we made several interesting observations regarding the lipid profile shifts within the tomato lines subjected to cold temperature conditions.

We first investigated the lipid composition in a tomato mutant line deficient in the gene of *ELONGATED HYPOCOTYL 5* (*HY5*), which serves as a vital regulator that integrates light, hormone, and stress signaling pathways to orchestrate plant responses to cold stress.^[^ ^8^ ^]^ HY5 can activate both C‐repeat/drought‐responsive element binding factor (**CBF)‐dependent and independent pathways,** which are crucial for cold adaptation. Additionally, HY5 has been found to enhance reactive oxygen species (**ROS) scavenging** and antioxidant defenses, alleviating oxidative damage induced by cold stress. Its regulatory capability extends to hormonal signaling, where HY5 can modulate abscisic acid (ABA) and jasmonic acid (JA) signaling pathways to optimize stress responses. Moreover, HY5 can suppress plant growth under cold conditions, thereby redirecting energy and resources away from developmental processes and toward stress adaptation mechanisms to enhance survival.^[^ ^29^, ^30^ ^]^ Under cold stress, slhy5 mutants showed reduced SFA, MUFA, and PUFA levels compared to WT. Our advanced MS approach revealed that conventional FA profiling misses critical lipid complexity, especially isomer‐specific variations. We further demonstrated that SlHY5 regulates cold‐responsive genes, FATTY ACID DESATURASES (FADs), which can facilitate the unsaturation of FAs. CRISPR‐generated slfad mutants mirrored slhy5 FA profiles, and exogenous isomer application partially rescued *slfad* phenotypes, offering practical applications. Overall, this advanced technology has revealed a fascinating dimension of the complex mechanisms that tomato plants utilize to cope with cold stress. The knowledge gained from this technology can serve as a foundation for future exploration of stress response mechanisms in other plants, broadening the scope of plant lipidomics and its applications in agriculture.

## 2 Results

### 2.1 Uncovering new Plant Lipid Species using Charge‐Tagging PB Derivatization and RPLC‐MS/MS

Understanding the structural diversity among lipid molecules can help unfold the complexities of cold stress tolerance in plants. FAs play a crucial role in numerous biological processes and they are vital components of cell membranes, energy storage, and signaling pathways.^[^ ^31^, ^32^, ^33^ ^]^ Despite the extensive research and cataloging of FAs that have occurred over the years, it is noteworthy that a significant number of naturally occurring FA molecules in plants remain unidentified and uninvestigated.^[^ ^34^, ^35^ ^]^ Historically, the investigation of FAs in plants has focused on a limited range of well‐characterized lipid compounds, which has inadvertently marginalized a vast array of uncharacterized FAs. This tendency is largely rooted in the prioritization of FAs that are readily identifiable and have established biological roles. As a result, many potentially significant FAs that may play crucial roles in plant physiology, development, and abiotic stress responses remain underexplored. The limited availability of efficient detection technologies is also another reason that has hindered the study of plant lipids comprehensively.^[^ ^36^ ^]^ To solve this, we implemented a sensitive and reliable workflow that combines charge‐tagging PB derivatization and RPLC‐MS/MS to precisely pinpoint and reveal C═C within lipids of the plant. This technology can identify unknown FAs even in cases where synthetic standards are not readily available.

**Figure 1:**
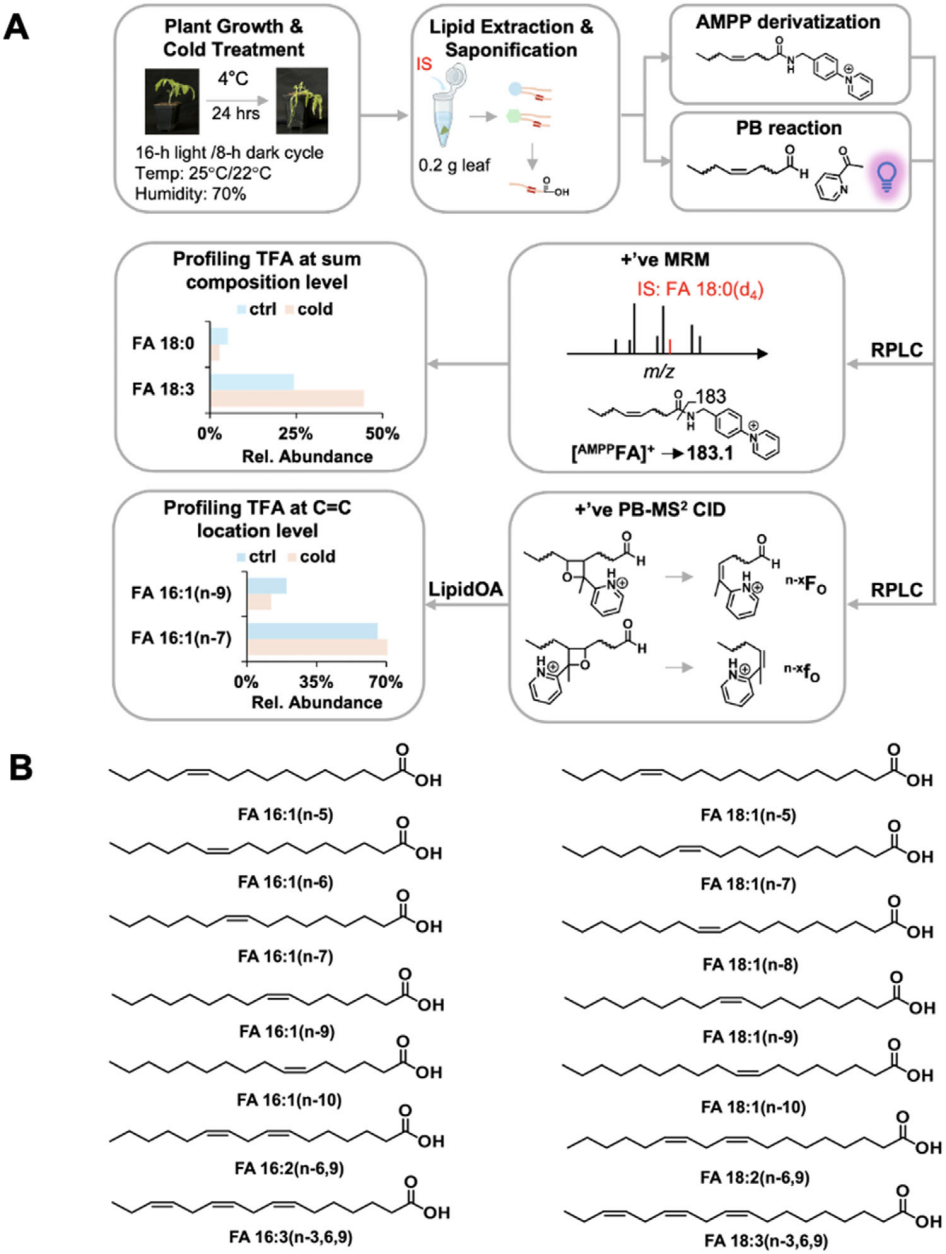
Analytical workflow for lipid analysis and identification in tomato plants. A) Tomato plants were grown until four weeks old before being subjected to cold stress conditions at 4 °C. Following 4 °C exposure, standard plant lipid extraction methods were employed to isolate the plant lipids. The extracted lipids underwent AMPP derivatization, PB reaction before getting analyzed by reversed phase liquid chromatography coupled with tandem mass spectrometry. These procedures enhanced the sensitivity and accuracy for the relative quantitation of total FAs at both the sum composition and C═C location levels. B) Identification of FAs including their C═C location isomers that can contribute to the cold tolerance of tomato plants by methodology presented in A.

In addition to its high sensitivity, the technology excels in its strengths of detecting synthetic standards in the sub‐nM range and performing relative quantitation of low abundance C═C location isomers at concentrations as low as 1% relative to the most abundant isomer.^[^ ^37^, ^38^ ^]^ Such precision in measurement is paramount for our lipidomics studies concerning plant cold stress tolerance because slight variations in a lipid structure can yield profound implications for plant physiology and stress response. To facilitate FA quantification, a dual offline derivatization process is used. In the first step, we performed AMPP derivatization and used MRM transition from [^AMPP^FA]^+^ to *m/z* 183.1, a characteristic AMPP fragment peak, to quantify both SFAs and UFAs at the chain composition level.^[^ ^26^, ^39^ ^]^ For the identification and quantification of C═C location isomers in UFAs, 2‐acetyl pyridine (2‐acpy) PB derivatization was performed. The PB reaction involves a [2+2] cycloaddition where 2‐acpy's carbonyl group reacts with a lipid's double or triple bond, forming a cyclopropane ring under UV light. Subsequent collision‐induced dissociation (CID) cleaves the resulting oxetane ring, generating diagnostic ions specific to the original C═C bonds.^[^ ^38^, ^40^, ^41^, ^42^ ^]^ This enhancement improves FA quantitation sensitivity and specificity, allowing for detailed lipid profiling in plant samples (**Figure** **1**A).

### 2.2 Probing for Fatty Acid Dynamics in Tomato Plants under Cold Stress

**Figure 2:**
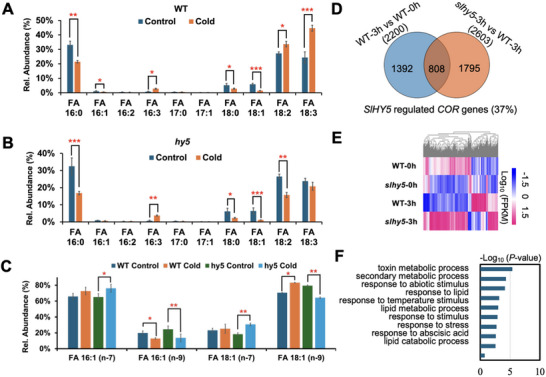
Fatty acid profiles of WT versus *slhy5* tomato mutant lines and genome‐wide identification of genes regulated by HY5 before and after cold treatment. Relative quantitation of FAs and their isomers at A) the sum composition and B) C═C levels in WT versus *slhy5* before and after cold treatment. C) Venn diagrams showing DEGs regulated by *Sl*HY5. D) Heatmap representing the DEGs regulated by *Sl*HY5. E) GO analysis indicating the top ten enrichment categories of *Sl*HY5‐regulated COR genes. F) Heatmap showing the well‐known COR genes in the WT and *slhy5* mutants. G) Heatmap revealing the fatty acid‐related genes in the WT and *slhy5* mutants. Values are means ± SD from three independent replicates. Statistical significance was determined by using the two‐tailed Student's *t‐*test (*P < 0.05, **P < 0.01, ***P < 0.001).

FAs are reported to play a significant role in the cellular response of plants to cold stress.^[^ ^43^, ^44^ ^]^ Plants undergo a series of physiological changes to adapt and survive the challenging conditions when exposed to low temperatures. One key mechanism is through the modulation of FA composition within the cell membrane of plants.^[^ ^45^, ^46^ ^]^ To confirm this, we grew WT and *slhy5* mutants of tomato plants and extracted FAs from the plant leaves after cold exposure using an established plant lipid extraction protocol (Figure 1A). We selected *slhy5* mutants for our test because *Sl*HY5 is a crucial regulator that can integrate light signaling to coordinate the stress response of plants. In tomato, *Sl*HY5 has been shown to directly activate *Sl*BBX31, which is a transcription factor that binds to the promoters of cold‐responsive *Sl*CBF genes, thereby stimulating their transcription and the downstream expression of cold tolerance genes.^[^ ^8^ ^]^ SFAs such as FA 16:0 and 18:0 and UFAs with one C═C (FA 16:1 and 18:1) significantly decreased under cold conditions in both the WT and *slhy5* mutants. UFAs with two and three C═Cs, such as FA 18:2 and 18:3, were observed to increase in the WT after cold treatment. However, FA 18:2 decreased in the *slhy5* mutants after cold exposure (**Figure** **2**A,B). When charge‐tagging PB derivatization and RPLC‐MS/MS were applied to further analyze these UFAs, remarkable insights into the behavior of FA isomers emerged. Particularly, this unique technology revealed the presence of previously undocumented isomers of mono‐unsaturated FAs (MUFAs), including FA 16:1(n‐5), FA 16:1(n‐6), FA 16:1(n‐7), FA 16:1(n‐9), and FA 16:1(n‐10) for FA 16:1; FA 18:1(n‐5), FA 18:1(n‐7), FA 18:1(n‐8), FA 18:1(n‐9) and FA 18:1(n‐10) for FA 18:1 (Figure 1B). We also identified the C═C location for polyunsaturated FAs (PUFAs), including FA 16:2(n‐6,9), FA 16:3(n‐3, 6, 9), FA 18:2(n‐6,9) and FA 18:3(n‐3, 6, 9). In particular, we saw a significant decrease in the FA 16:1(n‐9) for both the WT and *slhy5* mutants after cold exposure. FA 18:1(n‐9) was noticed to increase in the WT but decrease in the *slhy5* mutants. It was also interesting to observe an increase in their isomer forms, FA 16:1(n‐7) and 18:1(n‐7), in the *slhy5* mutants after cold exposure (Figure 2C). These findings indicated not only the complexity of lipid biochemistry but also the potential for undiscovered metabolic pathways in tomato plants under cold conditions. The differentiation of isomers is important as it may impact the functional properties of the FAs, influencing their roles in cellular structures, energy storage, and signaling processes. The lipidomics tools utilized in our current work offer a unique advantage by facilitating the identification of isomeric forms that would otherwise be eluded if using only conventional methods.

### 2.3 Deciphering the Complex Landscape of Fatty Acids and Isomer Formation under Cold Stress

The identification of new FA isomers opens exciting routes for further investigation, particularly concerning their biosynthetic mechanisms and functional roles under cold stress. The formation of isomers in FAs can be explained by the diverse enzyme systems that facilitate their synthesis through various pathways, such as *de novo* FA synthesis and elongation or desaturation of existing FAs.^[^ ^47^, ^48^ ^]^ Hence, FA biosynthesis is a multifaceted process that leads to the production of various types of FAs, including SFAs, MUFAs and PUFAs. Each of these FAs possesses unique structural and functional properties that contribute to their biological significance. In particular, we wanted to further understand the existence of various structural isomers in MUFAs under cold stress in tomato plants. The FA isomers observed in our study likely arose due to the diverse metabolic pathways involved in FA synthesis and the need for tomato plants to adapt to cold conditions.

To reveal key enzymes responsible for isomer formation in the FA synthesis pathway under cold conditions, we subsequently embarked on a comprehensive RNA sequencing study. By comparing the transcriptome profiles between WT and *slhy5* mutants under normal and cold stress conditions, we identified specific changes in the expression of cold‐regulated (COR) genes regulated by *SlHY5*. 2200 and 2603 genes were identified as COR genes in the WT and *slhy5*, respectively (Figure 2D). Of these, 808 genes overlapped and we classified them as SlHY5‐regulated COR genes, accounting for ≈37% of the COR genes and they displayed differentially expressed patterns between WT and *slhy5* as indicated on the heatmap (Figure 2D,E). Gene Ontology (GO) enrichment analysis further established that these HY5‐regulated COR genes are mainly enriched in the categories of toxin metabolic process, secondary metabolic process, response to abiotic stimulus, response to lipid metabolic processes as well as lipid catabolic process (Figure 2F). We selected several well‐known COR genes including *SlCBF1* and *SlCBF2* to examine the reliability of our RNA‐sequencing data and we found that their expressions were up‐regulated by cold stress in both the WT and *slhy5* mutants (Figure S1A, Supporting Information). Furthermore, the data facilitated the identification of fatty acid desaturases (*SlFAD*s) for their role in modulating FA synthesis and involvement in the formation of isomers (Figure S1B, Supporting Information). *Sl*FADs have been recognized for their enzymatic functions in FA metabolism but their role in isomer formation has not been extensively documented in plant research.^[^ ^49^, ^50^ ^]^ Our data indicated that *Sl*FAD expression levels exhibited significant alterations under cold, especially two putative *Sl*FAD genes *SlFAD2‐4* and *SlFAD2‐7*, thus suggesting a previously unrecognized role of *Sl*HY5 in modulating the balance observed between various FAs and their isomers, potentially influencing the cold stress tolerance of tomato plants.

### 2.4 The Positive Role of *SlFAD2s* for Cold Stress Response

In higher plants, the main FA species are C16 and C18, representing roughly 30% and 70% of total FAs, respectively.^[^ ^51^ ^]^ These FAs have various saturation levels and generally display none to three C═Cs for the main species. The variations in saturation are very likely derived from FADs. FADs are essential for the regulation of lipid metabolism, enabling organisms to adapt to varying physiological conditions by modifying the FA profiles of their membranes and signaling molecules.^[^ ^52^ ^]^ They also play a vital role in the oxidation and breakdown of FAs to produce energy.^[^ ^53^, ^54^, ^55^ ^]^ Despite their established importance in lipid metabolism, our comprehension of their broader biochemical functions, particularly concerning metabolic regulation under cold exposure, remains incomplete. This lack of understanding extends to the nuanced processes of isomerization, which involves the structural transformation of FAs into different isomers that can significantly influence their metabolic pathways and regulatory mechanisms under cold stress. The modulation of FA C═C location isomers can affect membrane fluidity and cellular signaling pathways, which are vital for cellular adaptation during cold stress.

**Figure 3:**
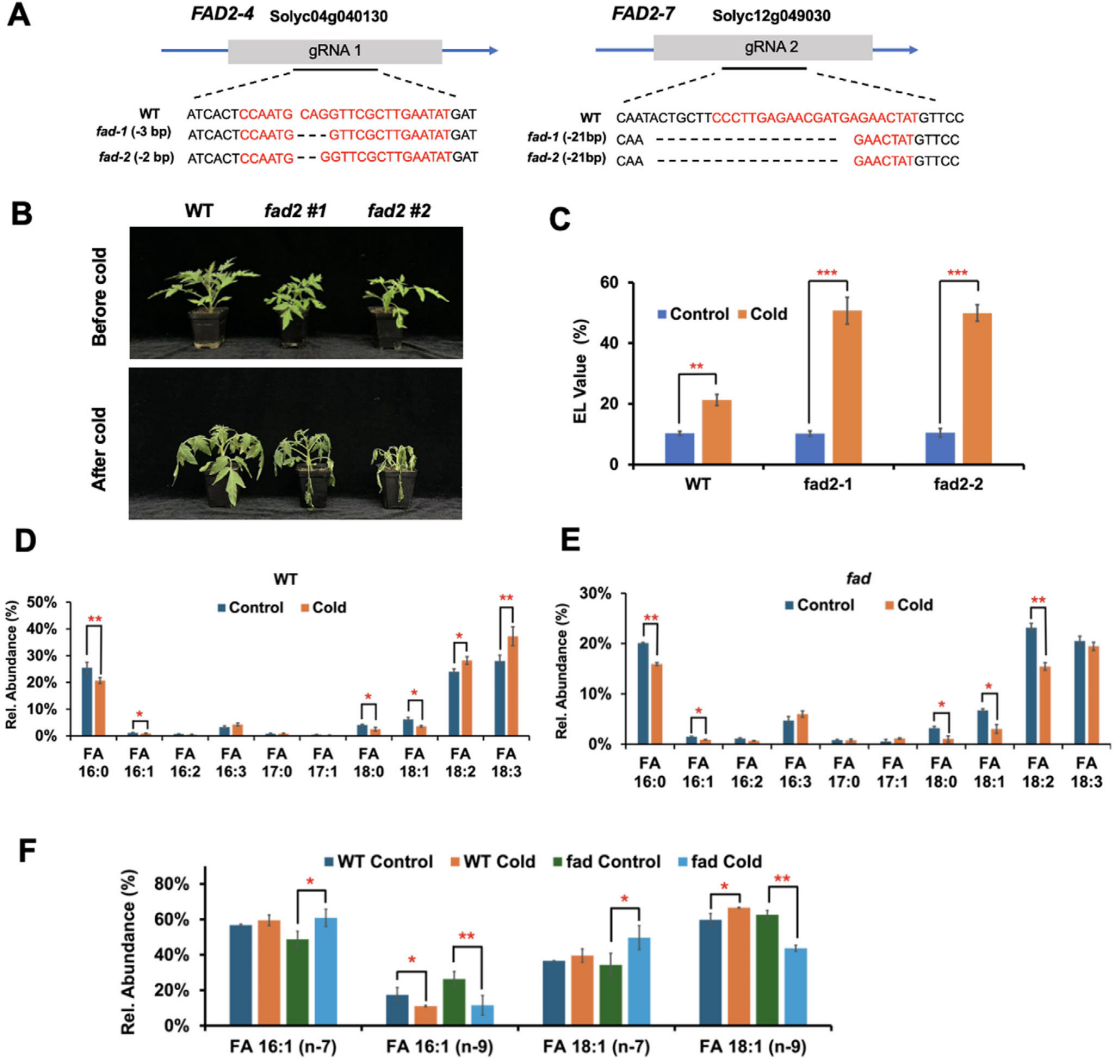
Construction and verification of *fad* tomato mutant lines and their lipid profiles. A) Schematic diagram of *slfad* CRISPR lines. B) The phenotypes of WT and *slfad* mutant lines in response to cold treatment. C) The electrolyte leakage values (%) of the WT and *slfad* mutant lines at room temperature (RT) and after cold exposure. Relative quantitation of FAs and their isomers at D) the sum composition and E) C═C levels in the WT versus *slfad* before and after cold treatment. Values are means ± SD from three independent replicates. Statistical significance was determined by using the two‐tailed Student's *t‐*test (*P < 0.05, **P < 0.01, ***P < 0.001), relative to WT.

To investigate the role of *Sl*FADs in isomerization formation, we designed two single‐guide RNA (sgRNA) targeting the two *SlFAD* genes (*SlFAD2‐4* Solyc04g040130 and *SlFAD2‐*7 Solyc12g049030) to generate the double mutants in the cultivar of Ailsa Craig (AC) using clustered regularly interspaced short palindromic repeats (CRISPR)‐associated protein 9 (Cas9; **Figure** **3**A). After screening tomato transformants, we isolated two mutant alleles in the AC background harboring 21‐bp deletions at the target sequence (Solyc12g049030) and 3‐bp or 2‐bp deletions at the target region (Solyc04g040130) (Figure 3A). We then subjected plants to cold conditions to assess their phenotypes and electrolyte leakage values. The *slfad* mutants exhibited a pronounced sensitivity to cold when compared to WT plants (Figure 3B,C). This finding aligned with previous studies that showed *slfad* mutants struggled to thrive under cold stress conditions. The augmented sensitivity observed in the *slfad* mutants indicates potential deficiencies in specific metabolic pathways or regulatory mechanisms that are integral to cold tolerance. To validate this, we subsequently utilized charge‐tagging PB derivatization and RPLC‐MS/MS to examine the FA profiles of plants. We observed that SFAs (FA 16:0 and 18:0) decreased in both the WT and the *slfad* mutants under cold conditions. UFAs such as FA 16:1 and 18:1 also decreased in both the WT and *slfad* mutants. On the other hand, FA 18:2 was observed to increase in the WT but decrease in the *slfad* mutants after cold exposure (Figure 3D,E). Upon closer examination of FA 16:1 and 18:1, we noticed a pattern of FA isomer changes similar to that observed in slhy5—where FA 16:1(n‐9) and FA 18:1(n‐9) levels decreased under cold exposure while isomers of FA 16:1(n‐7) and 18:1(n‐7) increased in the slfad mutants (Figure 3F). Such insights not only reinforced the importance of FA composition in plants for cold stress protection but also highlighted potential avenues for further research into the genetic and biochemical underpinnings of cold stress responses.

**Figure 4:**
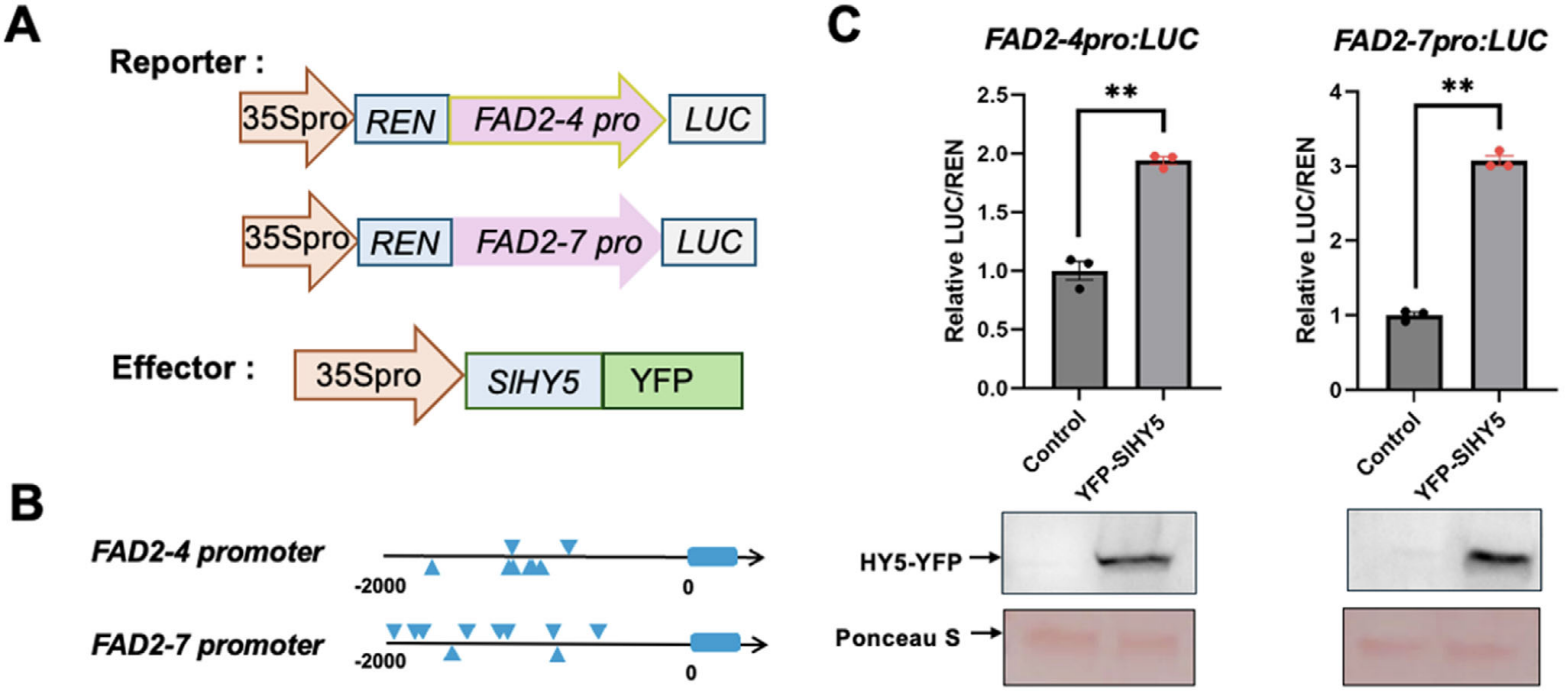
HY5 activates the transcription of *FAD2*s to induce the generation of unsaturated fatty acids and their isomers. A) Schematic diagrams of the effector and reporter constructs used for the dual‐LUC reporter assay. B) The potential HY5‐binding (ACGT) motifs in the promoter region of *SlFAD2‐4* and *SlFAD2‐7*. C) The relative LUC/REN value of *FAD2* promoter activities with or without HY5‐YFP. **P < 0.01, Student's *t*‐test, relative to WT. The protein abundance of effectors was detected by immunoblotting using an anti‐GFP antibody.

To determine whether the two *SlFAD2s* (*SlFAD2‐4* and *SlFAD2‐7*) are direct targets of SlHY5, we conducted dual‐luciferase (LUC) reporter assays. The promoters of  *SlFAD2‐4pro:LUC* and  *SlFAD2‐7pro:LUC* were used as reporters, while 35Spro:SlHY5‐YFP was co‐expressed as an effector (**Figure** **4**A,B). Co‐expression of *SlHY5‐YFP* significantly enhanced LUC activity from both reporter constructs, indicating that SlHY5 may directly regulate SlFAD2 expression (Figure 4C).

### 2.5 Understanding the Physiological Responses of Tomato Plants to Lipid Stimuli

**Figure 5:**
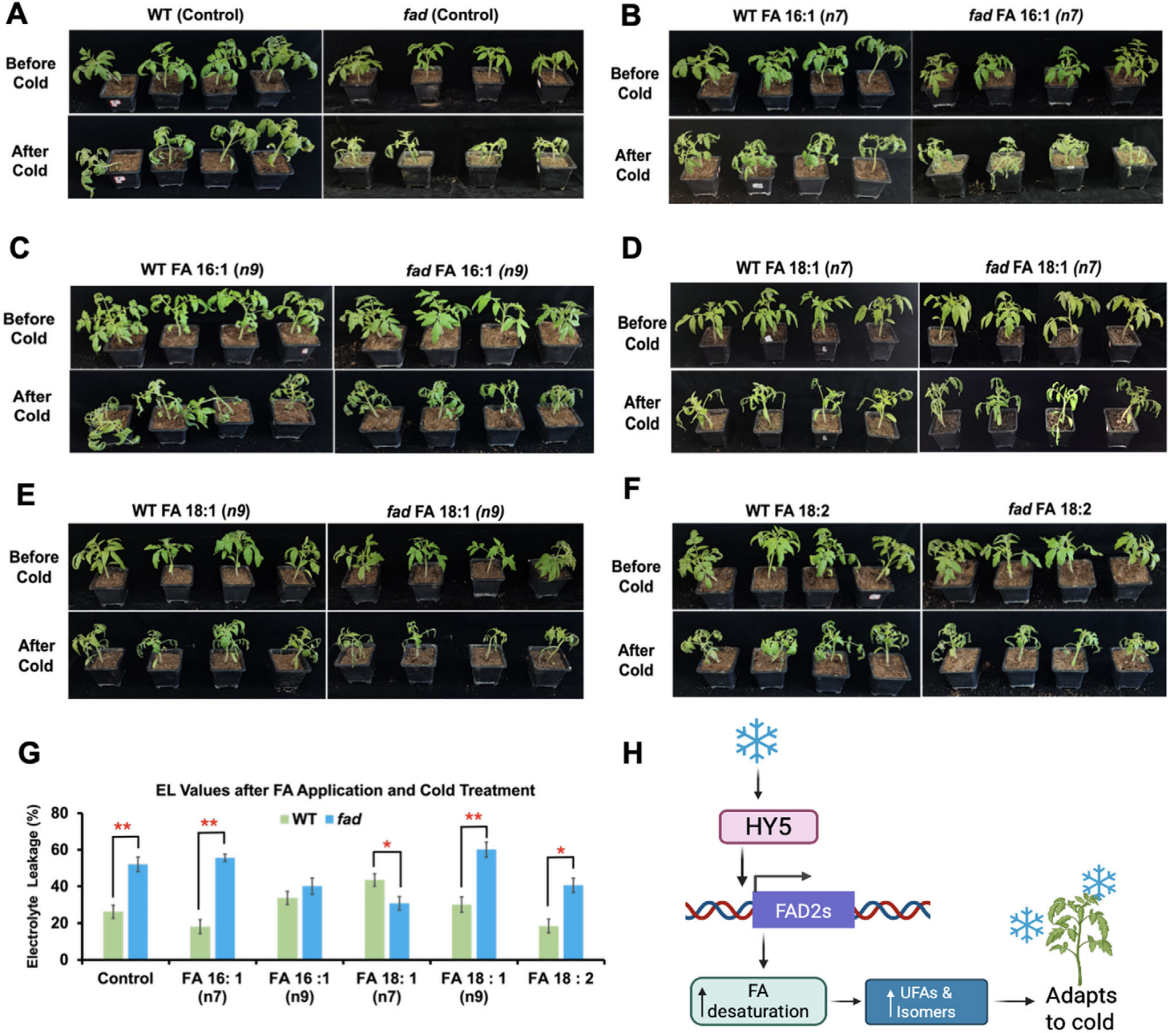
The phenotypic plasticity of WT and *slfad* in response to cold after fatty acid application. Phenotypes observed in WT and *slfad* mutant lines after the application of A) Control B) FA 16: 1(n‐7) C) FA 16:1(n‐9) D) FA 18:1(n‐9) E) FA 18:1(n‐7) F) FA 18:2(n‐6, n‐9) G) Electrolyte leakage values in the WT and *slfad* after applying FA and cold treatments. H) Proposed model for HY5‐mediated activation of FADs to generate lipids that enhance cold tolerance in tomato. Values are means ± SD from three independent replicates. Statistical significance was determined by using the two‐tailed Student's *t‐*test (*P < 0.05, **P < 0.01), relative to WT.

Phenotypic analysis following FA application is critical for elucidating the biochemical and physiological responses of organisms to lipid stimuli. Aside from serving as the fundamental components of cellular membranes and key signaling molecules, FAs can also significantly influence various metabolic pathways and physiological functions.^[^ ^56^ ^]^ By systematically examining the phenotypic changes after FA application, we can gain insights into how WT and *slfad* mutants adapt to cold stress responses in the presence of specific lipids. We have observed that both SFAs and UFAs, including their isomers, can play a significant role in the cold stress responses of tomato plants. Given the increasing frequency of cold events due to climate change and the fact that tomato plants are susceptible to cold stress, understanding how these FAs and their isomers contribute to their physiological and biochemical adjustments is vital for improving cold tolerance and overall plant resilience. Hence, we selected FAs based on MS analyses, specifically FA 16:1(n‐7), FA 16:1(n‐9), FA 18:1(n‐7), FA 18:1(n‐9) and FA 18:2(n‐6, n‐9) to investigate the phenotypic responses that could mitigate damage caused by low temperatures in tomato plants. Our analysis revealed intriguing insights into the effects of FAs on the cold tolerance of WT plants and *slfad* mutants. We found that the application of FA 16:1(n‐7) and FA 18:2(n‐6, n‐9) could improve the cold tolerance of WT plants when compared to the control (**Figure** **5**A,B,F). This finding suggested that these FAs may be essential for strengthening the membrane fluidity and general robustness of WT plants in cold climates. The structural properties of these FAs, which may better support the lipid bilayer of plant cells under stress, may be the mechanisms underlying this enhanced cold tolerance. *slfad* mutants, however, could adapt to cold better relative to the control when FA isomers of FA 16:1(n‐9) and FA 18:1(n‐7) were applied (Figure 5A,C,E). This difference indicated that the unique metabolic pathways and adaptations present in *slfad* mutants enabled them to respond more favorably to isomer application compared to their WT counterparts. FA 18:2(n‐6, n‐9) also appeared to offer some benefits to the *slfad* mutants relative to their control (Figure 5A,F). FA 18:1(n‐9) appeared to hinder the ability of both the WT and *slfad* mutants to resist cold temperatures (Figure 5D).

We also explored the effects of FAs and their isomers on electrolyte leakage (EL) in the WT and *slfad2* mutant plants. EL, which measures the loss of ions and other cellular contents from plant tissues, is an indicator of cellular damage and membrane integrity.^[^ ^57^, ^58^ ^]^ Our data demonstrated that treatments of FA 16:1(n‐7) and FA 18: (n‐9) isomers increased EL in the *slfad* mutants relative to their control, suggesting a potential stress response resulting from the isomer treatments. Meanwhile, treatments of FA 16:1(n‐9) and FA 18:1(n‐7) increased EL of the WT more (Figure 5G). This increase in leakage could indicate compromised membrane integrity and enhanced permeability, often associated with oxidative stress or cellular damage from cold. In contrast, the same treatments applied to the *slfad* mutant plants led to a decrease in EL, suggesting a type of resistance or improved membrane stability (Figure 5G). This differential response highlighted the potential adaptive mechanisms of the *slfad* mutants to lipid modifications and stress cues. The differential response observed in *slfad* mutants compared to WT plants upon the application of various FA isomers highlights the intricate nature of plant responses to environmental stress and this insight could pave the way for developing strategies to improve crop resilience in changing climate conditions.

## 3 Discussion

Plant FAs play a pivotal role in how plants respond to and manage different environmental stresses. In higher plants, FAs with 18 carbon atoms represent ≈70% of the total FAs, with the unsaturated varieties: oleic acid (18:1 (n‐9), linoleic acid (18:2 (n‐6, n‐9) and alpha‐linolenic acid (18:3(n‐3, n‐6, n‐9) being the most prevalent.^[^ ^48^, ^59^ ^]^ This high abundance highlights the significance of UFAs in plant physiology. Rising studies recognize UFAs as strategic players in the plant's arsenal against diverse stress factors, including cold exposure. UFAs are ubiquitously found in terrestrial plants and they have been associated with mechanisms that help mitigate the adverse effects of abiotic stresses such as temperature changes.^[^ ^60^, ^61^ ^]^ In plants, UFAs 18:1, 18:2, and 18:3 contribute to membrane fluidity and can act as precursors for signaling molecules that activate defense pathways under cold stress.^[^ ^48^ ^]^ Despite the evident significance of these UFAs in the context of cold stress, research exploring the specific roles of UFAs and their isomers in cold stress response remains scarce. Therefore, understanding the intricate relationships between FA composition, isomer variations, and cold stress responses in plants is a significant frontier for agricultural practices and crop resilience in changing climates.

Studying lipids, however, poses significant challenges due to their complex structures. FAs are a class of lipids that consists of a vast array of saturated and unsaturated forms, each tailored for specific physiological roles. The intricate nature of lipid molecules, including their nonpolar characteristics and diverse arrangements, makes traditional analytical techniques insufficient for comprehensive plant lipid characterization.^[^ ^62^, ^63^, ^64^ ^]^ As a result, there is a need for advanced methodologies that can effectively unravel the structural complexities of plant lipids. Our methodology combines PB photochemistry with LC‐MS/MS to precisely map lipid structures. The PB reaction uses UV‐activated [2+2] cycloaddition between 2‐acpy carbonyl groups and lipid C═C bonds, forming oxetane rings that yield diagnostic fragments during CID‐MS. This approach uniquely identifies UFA isomers by their original C═C positions. Charge‐tagged PB derivatization enables sensitive, high‐resolution lipid profiling, thus revealing structural features of lipid classes and isomers beyond conventional plant lipid analysis.

As the essential building blocks that compose lipids found within the cell membranes of plants, FAs play a critical role in maintaining the fluidity and structural integrity of the membrane system in plants. When plants sense environmental stress, they can adapt by altering the composition and ratios of the different FAs present in their membranes. Leveraging this natural mechanism, we have utilized WT, *slhy5*, and *slfad* mutant plants to further explore and understand the intricate relationships between FAs and plant stress responses. Our findings indicated that upon exposure to cold temperatures, the levels of SFAs decreased in both the WT and mutants. Cold stress induced distinct alterations in UFAs and their isomers. These changes suggested a strategic response utilized by the plants to regulate their membrane fluidity and maintain cellular functionality under low‐temperature conditions, as indicated by some reports.^[^ ^65^, ^66^, ^67^ ^]^ UFAs possess distinctive kinks and bends in their hydrocarbon chains in contrast to the rigid, straight‐chain structure of SFAs. The rigid, linear SFA molecules tend to pack together tightly that can cause the cell membranes to stiffen and potentially rupture in the cold. On the other hand, the kinked shape allows the UFA molecules from solidifying and crystallizing even in the face of freezing temperatures. Therefore, the molecular architecture of UFAs grants them a greater degree of flexibility and adaptability as temperatures begin to drop. The coordinated changes in SFA and UFA levels demonstrated the remarkable ability of plants to rapidly reprogram their lipid metabolism to optimize membrane properties and bolster their cold tolerance.

In addition, we observed significant ratio changes of isomers, particularly n‐7 and n‐9, in FA 16:1 and FA 18:1, which was intriguing as they exhibited some effects on the response to cold in tomato plants. The isomers of FA 16:1(n‐9) and FA 18:1(n‐9) decreased while 16:1(n‐7) and 18:1 (n‐7) increased upon cold exposure in the mutant lines. Meanwhile, we noticed the isomers of FA 16:1 (n9) decreased while FA 18:1 (n9) increased in the WT under cold conditions. This divergent response implied that the n‐9 and n‐7 isomers are intricately involved in the cold‐adaptation mechanisms. Their changes could indicate a shift in metabolic priorities or the diversion of resources toward the production of other isomers, PUFAs, and cold‐protective compounds. The intricate interplay between various FAs has been demonstrated to play a significant role in the cold response mechanism of tomatoes. From our study, *Sl*HY5 appeared to have the ability to orchestrate the expression of *Sl*FADs, which are involved in the synthesis and modification of FAs. This regulation can drive the production of specific FA isomers and increase PUFAs such as FA 18:2 and 18:3, contributing to the cold tolerance of tomato plants as proposed in Figure 5H. The collaboration between diverse FAs, which *Sl*HY5 and *Sl*FAD can mediate, further exemplifies the sophisticated adaptive strategies plants employ to fine‐tune biochemical pathways for survival and growth under suboptimal conditions.

In the realms of agriculture and biotechnology, the practice of phenotypic analysis is critical for the success of breeding programs and the development of crops that exhibit enhanced resilience against various environmental stresses.^[^ ^68^, ^69^ ^]^ By closely examining the specific physiological and morphological responses of tomato plants to the application of different FA compounds, we could gain invaluable insights that inform the optimization of lipid compositions to amplify desirable traits. We observed that the applications of commercially available isomers (FA 16:1(n‐9) and FA 18:1(n‐7) on the leaves of the *slfad* mutants could partially rescue their cold sensitivity while FA 16:1(n‐7) and FA 18:2(n‐6, n‐9) appeared to be more beneficial for the WT to resist cold. The *slfad* mutant plants displayed reduced signs of leaf curling and wilting compared to their control after being treated by the isomers of FA 16:1(n‐9) and FA 18:1(n‐7). The EL data served as a crucial complement to the phenotypic analysis, providing deeper insights into the impacts of the various FA treatments on the plants. When the WT plants were subjected to treatments with FA isomers of FA 16:1(n‐9) and FA 18:1(n‐7), their EL values increased. The elevated EL in the WT plants suggested that the introduction of these FA isomers disrupted the normal functioning and permeability of the cell membranes, potentially leading to a compromised ability to retain essential electrolytes and other important cellular components. In contrast, the same FA isomer treatments resulted in a decrease in EL within the mutant plants. This inverse response implies that these mutant plants somehow conferred an enhanced resilience or adaptability due to the presence of these FA isomers, allowing the mutant lines to better maintain their membrane structures and electrolyte balance.

The stark difference in how the WT and *slfad* mutant plants responded to the FA isomer treatments underscores the complex interplay between lipid composition, membrane properties, and plant stress responses. Exploring the intricate connections between lipid composition, membrane function, and stress physiology is a crucial area of future research that warrants deeper investigation. The powerful platform of PB‐LC‐MS/MS represents an advanced analytical tool that enables us to investigate deep into the complex world of lipids and their dynamic response to cold stress within tomato plants. This improved technology can provide a rich, high‐resolution dataset that paints a comprehensive picture of diverse lipid species, and not just FAs, in the plant samples. By leveraging the FA information generated from this platform, we strategically extrapolated and applied targeted FA compounds that showed the capacity to enhance the stress tolerance of tomato plants. While our MS platform enables precise lipid identification at the molecular level, combining these results with atomic force microscopy and Laurdan generalized polarization fluidity measurements in future studies could provide a more comprehensive understanding of membrane reorganization during stress responses. Therefore, our work is an important step forward in leveraging advanced analytical technologies to unlock the answers to plant stress biology and translate those insights into practical solutions for the benefit of agriculture.

## 4 Experimental Section

### Plant Materials and Growth Conditions

The Ailsa Craig (AC, LA2838A) cultivars were used as the wild‐type (WT) in this study. The WT and mutant plants were grown under long‐day (LD) condition with 16‐h light /8‐h dark cycle, 25 °C during the day and 22 °C at night, 70% humidity.

### Construction of CRISPR‐Cas9 Vectors and Generation of Tomato Mutant Lines

The CRISPR‐Cas9 vectors were constructed as previously described.^[^ ^8^, ^9^ ^]^ Primers used for the construction of CRISPR vectors and screening of transgenic plants for gene editing are listed in Table S1 (Supporting Information). Following the transformation of tomato cultivars at Weimi company, a thorough screening process using at least 30 T_0_ transformants were performed to identify those that exhibited the desired genetic modifications at the targeted sites. After a careful selection and evaluation, the T_3_ homozygous lines (Cas9‐free) were isolated and used for subsequent analyses.

### Cold Stress Treatment and Electrolyte Leakage Measurement

For cold stress treatment, tomato plants (4‐week‐old) were placed in a cold room (4 °C) for 24 h before lipid extraction. To study the phenotypic responses of tomatoes to the application of fatty acids, 100 µL of each fatty acid (>95% purity, Cayman Chemical Company) was withdrawn and placed into a 30 mL of water:ethanol mixture (99.5%/0.5%,V/V). The cold tolerance was then recorded after 24 h in the cold room. The electrolyte leakage (EL) was measured as described in (Zhu et al., 2023). The EL measurements were repeated at three independent times.

### Lipid Extraction and Sample Preparation

Total lipids were extracted from 0.2 g of tomato leaves with FA 18:0(D4) (10 µm) added as an internal standard according to previous reports^[^ ^18^, ^19^ ^]^ Tomato leaves were immersed in 3 mL of 75 °C isopropanol with 0.01% butylated hydroxytolune for 15 min. After cooling to room temperature, 1.5 mL chloroform and 0.6 mL water were added and gently shaken overnight. Extracts were carefully removed and re‐extracted four times with 4 mL chloroform:methanol (2/1, v/v) with 0.01% BHT and 30 min of shaking each time. All extracts from one leaf were combined, dried, and weighed. Then, 0.2 mg of lipids were saponified in 500 µL ACN:15% NaOH (50/50, v/v) at 60 °C for 30 min. The solution was acidified by 4 m HCl to adjust the pH ≈5. The hydrolyzed lipids were extracted twice with 1.5 mL MTBE each time. The organic layer was collected, dried under nitrogen, and redissolved in an aliquot of 200 µL MeOH for further derivatization. FA 18:0(D4) and AMP^+^ kit were purchased from Cayman Chemical (Ann Arbor, MI). PB reagent, 2‐acpy were purchased from BidePharm (Shanghai, China). Isooctane, methyl tert‐butyl ether, HPLC grade acetonitrile (ACN), methanol (MeOH), isopropanol (IPA) were purchased from Fisher Scientific Company (Ottawa, ON, Canada).

### AMPP Derivatization

AMPP derivatization was carried out according to the protocol specified in the AMP^+^ MaxSpec Kit. Lipid extracts in 50 µL of solution were dried under a nitrogen stream and subsequently redissolved in a mixture containing 10 µL of a 4:1 acetonitrile/dimethylformamide (ACN/DMF) solution, 10 µL of 1‐ethyl‐3‐(3‐dimethylaminopropyl) carbodiimide (EDC) (640 mm in H2O), 5 µL of N‐hydroxybenzotriazole (HOBt) (20 mm in a 99:1 ACN/DMF solution), and 15 µL of AMPP (20 mm in ACN). This mixture was incubated at 60 °C for 30 min. Upon cooling to room temperature, 600 µL of water was added to the solution. AMPP derivatized sample was extracted twice by 600 µL methyl tert‐butyl ether (MTBE) and dried under a nitrogen stream. For LC‐MS/MS analysis, the dried sample was reconstituted in 125 µL of MeOH. To determine the concentration of each fatty acid (FA) in the sum composition, multiple reaction monitoring (MRM) transitions from [^AMPP^FA]^+^ to *m/z* 183.1—a characteristic AMPP fragment peak—were used to quantify both saturated and unsaturated FAs at the chain composition level.

### Offline PB Reaction

The PB derivatization was conducted using a custom‐built flow microreactor.^[^ ^38^, ^39^ ^]^ FA extracts, initially dried from a 150 µL solution and mixed with a 10 mm 2‐AcPy reagent, were redissolved in 100 µL of ACN. This solution was then subjected into the flow microreactor for 20 s of UV irradiation (λ = 254 nm), then 50 µL of the reaction solution was collected. Due to the unavailability of synthetic standards for most C═C location isomers found in biological samples, a relative quantitation approach for isomer composition was employed. This involved summing the peak intensities of the C═C diagnostic ions (^n‐x^F_O_ and ^n‐x^f_O_) as ∑ I_n−x_. The relative composition of each ^n‐x^C═C isomer was then calculated using the formula: ∑ I_n−x_ / (∑ I_n−x_ + ∑ I_n−y_ + ∑ I_n−z_…), where n‐y and n‐z represent other detected C═C location isomers from the same PB‐MS^2^ CID spectrum. In this study, the software LipidOA^4^ was utilized to perform the relative quantitation of C═C location isomers.

### LC‐MS

Reversed‐phase (RP) liquid chromatography‐tandem mass spectrometry (LC‐MS/MS) analysis were performed using a Shimadzu LC‐20AD system (Kyoto, Japan), coupled with an X500R QTOF mass spectrometer (Sciex, Toronto, Canada). The sample injection volume was set at 2 µL per run. A C18 column (150 mm × 3.0 mm, 2.7 µm, Sigma–Aldrich, MO) was used for separation. Mobile phase A consisted of H_2_O:ACN (40:60, v/v, with 20 mm ammonium formate) and mobile phase B comprised IPA:ACN (40:60, v/v, with 0.2% formic acid). The flow rate was maintained at 0.45 mL min^−1^. The chromatographic gradient was programmed as follows: 30% B for 0–0.75 min, a gradual increase from 30% to 45% B over 0.75–2 min, 45% to 52% B over 2–2.5 min, 52% to 58% B over 2.5–4 min, 58% to 66% B over 4–5.5 min, 66% to 70% B over 5.5–7 min, 70% to 75% B over 7–9 min, 75% to 97% B over 9–10 min, maintained at 97% B for 10–13 min, and returning to 30% B for 13.1–15 min. MS parameters were optimized as follows: electrospray ionization (ESI) voltage at 4500 V; curtain gas at 35 psi; interface heater temperature at 450 °C; nebulizing gases 1 and 2 set at 30 psi; declustering potential at 100 V; CID energy for PB‐MS/MS at 25 eV; and CID energy for multiple reaction monitoring (MRM) at 50 eV.

### Lipid Nomenclature

Shorthand notations of lipid structure followed the suggestions by LIPID MAPS.^[^ ^23^ ^]^ Taking FA 18:1(n‐9) as an example, this annotation specifies a fatty acid contains 18 carbons with one C═C bond the ninth carbon from the methyl terminus of the fatty acid chain.

### Dual Luciferase Reporter Assays

*SlFAD2‐4pro:LUC* and *SlFAD2‐7pro:LUC* Reporters were transiently co‐expressed with or without effector *SlHY5‐GFP* in Arabidopsis protoplasts as described.^[^ ^8^, ^9^ ^]^ After overnight transformation, the total protein was extracted and the LUC and REN signals were detected by BioTek Synergy H1 Microplate Reader. The protein expression of effector SlHY5‐YFP was detected with anti‐GFP antibody (11814460001) by western blot.

### Statistical Analyses

Statistical significance between two groups was assessed using a two‐tailed Student's *t*‐test, with P <0.05 considered significant. All experiments were performed with at least three biologically independent replicates (*n*≥ 3). Statistical analyses and the number of replicates for each experiment are indicated in the figures. In all figures: *: *p* < 0.05, **: *p* < 0.01, ***:*p* < 0.001.

## Conflict of Interest

The authors declare no conflict of interest.

## Author Contributions

L.C. and H.S. contributed equally to this work. LC. and Y.F.Z. designed and directed the project. L.C., H.S., Q.Y., X.S., Z.D., and Z.J. performed the experiments. L.C., H.S., Y.X., and Y.F.Z. analyzed the data and provided valuable comments. L.C. and H.S. drafted the manuscript; and L.C., H.S., Y.X., and Y.F.Z. revised the manuscript.

## Supporting information



Supporting Information

Supporting Information

Supporting Information

## Data Availability

The data that support the findings of this study are available from the corresponding author upon reasonable request.
