# Supplementary material for: A Mass Spectrometry Approach Reveals Fatty Acid Isomerism in Tomato Cold Tolerance
Source: Adv Sci (Weinh). 2025 Aug 4;12(40):e00175. doi: 10.1002/advs.202500175 (PMC12561362; doi:10.1002/advs.202500175)
Supplement: Supplementary file 1 — Supporting Information [file ADVS-12-e00175-s001.pdf]

Figure S1

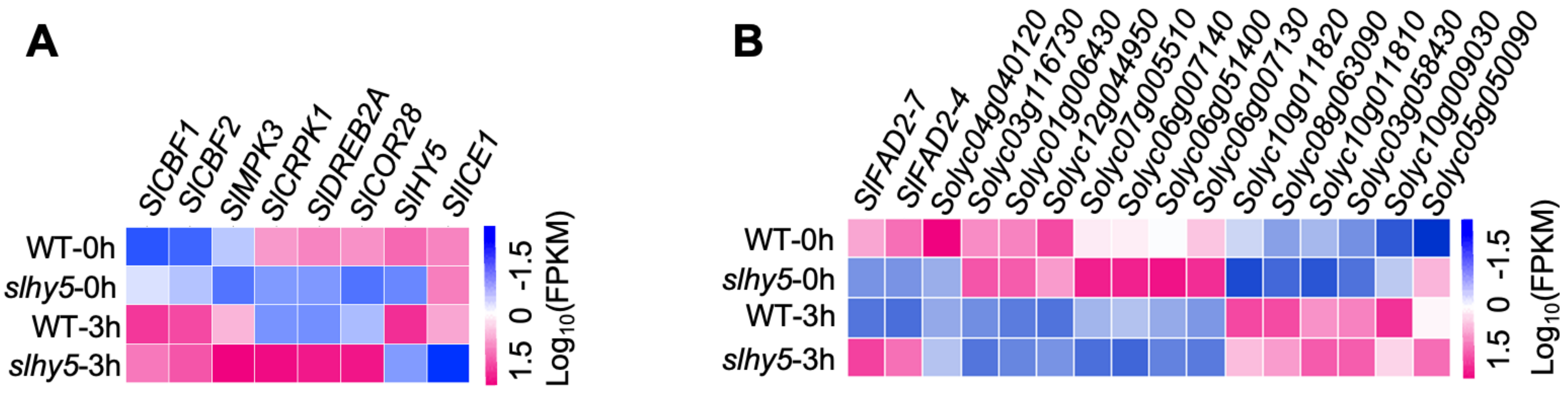

Figure S1. The differentially expressed COR and FAD genes identified from RNA-seq analysis.

A. Heatmap showing the well-known COR genes in the WT and *slhy5* mutants. B. Heatmap revealing the expression pattern of fatty acid-related genes in the WT and *slhy5* mutants.
